# Tuning contact line dynamics on slippery silicone oil grafted surfaces for sessile droplet evaporation

**DOI:** 10.1038/s41598-023-50579-2

**Published:** 2024-01-19

**Authors:** Astrid Raynard, Anam Abbas, Steven Armstrong, Gary G. Wells, Glen McHale, Khellil Sefiane, Daniel Orejon

**Affiliations:** 1 Institute for Multiscale Thermofluids, School of Engineering, The University of Edinburgh, Edinburgh, EH9 3FD Scotland, UK; 2 Department of Mechanical Engineering, University of Engineering and Technology, Lahore, 39161 Pakistan; 3 International Institute for Carbon-Neutral Energy Research (WPI-I2CNER), Kyushu University, 744 Motooka, Nishi-ku, Fukuoka, 819-0395 Japan

**Keywords:** Chemical engineering, Mechanical engineering

## Abstract

Controlling the dynamics of droplet evaporation is critical to numerous fundamental and industrial applications. The three main modes of evaporation so far reported on smooth surfaces are the constant contact radius (CCR), constant contact angle (CCA), and mixed mode. Previously reported methods for controlling droplet evaporation include chemical or physical modifications of the surfaces via surface coating. These often require complex multiple stage processing, which eventually enables similar droplet-surface interactions. By leveraging the change in the physicochemical properties of the outermost surface by different silicone oil grafting fabrication parameters, the evaporation dynamics and the duration of the different evaporation modes can be controlled. After grafting one layer of oil, the intrinsic hydrophilic silicon surface (contact angle (CA) ≈ 60°) is transformed into a hydrophobic surface (CA ≈ 108°) with low contact angle hysteresis (CAH). The CAH can be tuned between 1° and 20° depending on the fabrication parameters such as oil viscosity, volume, deposition method as well as the number of layers, which in turn control the duration of the different evaporation modes. In addition, the occurrence and strength of stick–slip behaviour during evaporation can be additionally controlled by the silicone oil grafting procedure adopted. These findings provide guidelines for controlling the droplet-surface interactions by either minimizing or maximising contact line initial pinning, stick–slip and/or constant contact angle modes of evaporation. We conclude that the simple and scalable silicone oil grafted coatings reported here provide similar functionalities to slippery liquid infused porous surfaces (SLIPSs), quasi-liquid surfaces (QLS), and/or slippery omniphobic covalently attached liquid (SOCAL) surfaces, by empowering pinning-free surfaces, and have great potential for use in self-cleaning surfaces or uniform particle deposition.

## Introduction

The manner droplets evaporate on solid surfaces is important in various applications such as pesticide spraying^1^, DNA mapping^2^, and thermal management processes like spray cooling^3^. Both experimental and theoretical studies have found that surface wettability^4^, roughness^5^, chemical heterogeneity^6^, the nature of the fluid^7^ and ambient conditions^8^, can cause the contact line of a droplet to behave as pinning-free, pinned, or lead to an uncontrolled combination of the two. Understanding the origin and cause of these behaviours allows the establishment of a surface’s suitability for specific applications and/or to engineer surfaces or coatings with desired evaporation dynamics. For example, a highly pinning surface may not be suitable for particle deposition where a uniform layer of particles is desired as it can lead to the well-known coffee-ring stain effect^9^.

To control the evaporation dynamics of droplets, it is important to understand how to modify a solid surface to tailor the interactions between the droplet contact line and the surface during evaporation, by either removing or imposing contact line pinning. In an ideal situation where the surface is completely flat and homogeneous, the shape of a small droplet in thermodynamic equilibrium can be approximated to a spherical cap, which can be described by the angle that the droplet makes with the surface known as the equilibrium contact angle (${\theta }_{{\text{e}}}$). The ${\theta }_{{\text{e}}}$ can be described by the Young's equation^10^, Eq. (1):

1 $${\text{cos}}{\theta }_{{\text{e}}}=\frac{{\gamma }_{{\text{SV}}}-{\gamma }_{{\text{SL}}}}{{\gamma }_{{\text{LV}}}}$$

where ${\gamma }_{{\text{SV}}}$ ,${\gamma }_{{\text{SL}}}$, and ${\gamma }_{{\text{LV}}}$ are the solid-vapour, solid–liquid and liquid–vapour interfacial tensions, respectively. A small finite liquid volume adopts a spherical cap shape on a surface when ${\gamma }_{{\text{SV}}}\ge {\gamma }_{{\text{SL}}}$ and the size of the droplet is below its capillary length, known as partial-wettability. Whereas when ${\gamma }_{{\text{SV}}} \ge {\gamma }_{{\text{SL}}}+{\gamma }_{{\text{LV}}}$, the liquid is drawn into a film across the surface with no observable contact angle and complete wetting occurs. The equilibrium contact angle measured for droplets on non-ideal surfaces varies due to physical and chemical heterogeneities of the surface, which typically induce droplet contact line pinning. The range of contact angles at which a droplet’s contact line can remain stationary as its volume is slowly increased or decreased on a non-ideal surface is defined by the contact angle hysteresis (CAH). This range of attainable contact angles lies between the advancing contact angle, *θ*_A_, and the receding contact angle, *θ*_R_. The advancing contact angle can be defined as the maximum contact angle that a droplet can achieve before the contact line advances over the surface. Whereas the receding contact angle is the minimum contact angle of droplet before the contact line recedes over the surface. The CAH is then defined as the difference between advancing and receding contact angle. The lower the CAH, the lower the pinning forces on the surface and hence the lower the droplet-on-surface static friction^11,12^. Therefore, reducing the CAH on a surface, while it remains an important challenge to be addressed, can bring benefits to droplet manipulation and control during microfluidics or droplet evaporation applications.

When droplets are gently deposited on solid surfaces, interfacial tensions, gravity, and capillary forces, drive the contact line spreading until equilibrium is attained^13^. Thereafter if the fluid is not in equilibrium with its vapour, evaporation occurs. The evaporation dynamics have been found to depend greatly on the droplet shape, the contact with the surface, and the surface properties. In 1924 Hedestrand reported that the substrate surface effects greatly the rate of evaporation of water drops vanishing either in the Constant Contact Radius (CCR) or in the Constant Contact Angle (CCA) mode^14^. In the CCR mode the contact angle decreases during evaporation while the contact radius remains constant, i.e., contact line pinning. Whereas in the CCA mode, the contact angle is expected to retain a constant value approximating the receding contact angle, *θ*_R_, while the droplet base radius or the base area decreases linearly in time. Thereafter, Picknett and Bexon proposed an analytical model that provides an exact solution for describing the ideal case in the presence of diffusion-limited evaporation of a sessile droplet in the absence of gravity for both of these modes^4^. The CCR mode typically occurs on hydrophilic high energy surfaces or coatings as reported by Rowan, et al. and a constant evaporative flux was observed during most of the droplet evaporation lifetime^15^. In the particular case of particle laden droplets on smooth surfaces, contact line pinning or CCR mode can result in a ring-like pattern being formed known as the “coffee-stain effect”^9^.

Surface modifications and coatings have been widely investigated to manipulate the interactions and modes of evaporation^16–19^. Low energy or hydrophobic surfaces or coatings allow for the CCA mode of evaporation to ensue^4,15,20^. In the CCA mode droplets typically evaporate following a *Volume*^2/3^ function of *time* relation^4,7,15,21^. Besides hydrophobic surfaces, Slippery Liquid Infused Porous Surfaces (SLIPSs), which are essentially a hydrophobic textured porous surface further impregnated with a lubricant oil, offer virtually no-pinning^22–24^ with occurrence of the CCA mode during most of the droplet evaporation lifetime as reported by Guan et al.^25^. The implementation of SLIPSs has been proved as an excellent strategy for removing contact line pinning; however, since the lubricant is mobile, eventually they may suffer from oil depletion limiting their effectiveness in broader industrial applications^26^. We also note here that the surface structure underneath the oil is of paramount importance to ensure pinning-free evaporation or the occurrence of discrete stick–slip events as in Üçüncüoğlu and Erbil^27^. Recent comprehensive reviews of sessile drop evaporation are given by Wilson and D'Ambrosio^28^, Erbil^29^, Cazabat and Guéna^30^, Larson^31^, Erbil and McHale^32^, and Wang et al.^7^.

By physical and/or chemical surface modification, both the wettability ${\theta }_{e}$ as well as surface heterogeneities associated to the CAH, can be tailored to our advantage depending on the application, empowering the ultimate control of the droplet evaporation dynamics^11,12,33^. It is the outermost substrate layer that governs the interactions between liquid droplets and the surface, rather than the internal bulk solid material. For example, a silicon wafer can be coated with a thin layer of a hydrophobic promoter such as Teflon modifying the intrinsic wettability of the surface from hydrophilic to hydrophobic. In addition, by changing the wettability of the original surface, the evaporation behaviour can be also modified. In the particular case of silicon and Teflon coated silicon, the pinning/CCR mode is reduced from 40% to approximately 4% while the CCA mode duration increases from 50 to 80% of the droplet lifetime^20^. Hence, the implementation of a thin coating, on an otherwise intrinsically hydrophilic surface, anticipates the control of these interactions making the surface underneath hydrophobic by deposition of low surface energy coatings or hydrophilic by implementing high surface energy ones^34^. Many techniques to improve the hydrophobicity and/or the low adhesion of surfaces and/or to fabricate hydrophobic coatings are well established^35–38^. To this end, on smooth hydrophobic surfaces, Orejon et al. reported an initial CCR mode for 10% of the evaporation, then 80% in the CCA and a final 10% in the mixed mode^20^. While on superhydrophobic pillared surfaces, Dash and Garimella reported an initial CCR mode for 20% of the evaporation, then 60% in the CCA and finally 20% in the mixed mode^39^. We note here that although the presence of pillars on a superhydrophobic surface should in turn impose a finite stick-slip behaviour as reported by Al Balushi et al., it may be the lack of spatial resolution the reason for the CCA mode instead stick-slip reported in Dash and Garimella^39,48^. Most recently, slippery omniphobic covalently attached liquid (SOCAL) and quasi-liquid slippery (QLS) surfaces have gained attention due to their low pinning ability, ease of contact line mobility and unprecedented liquid repellency^11,33,40^. Such surfaces can impose either hydrophilic or hydrophobic wettability with low contact angle hysteresis, empowering evaporation in the CCA mode during most of the droplet lifetime on a smooth flat surface^21,25,41^. In addition to SOCAL and QLS surfaces, Slippery Liquid Infused Porous Surfaces (SLIPSs) have also been reported to show low CAH with droplets evaporating in the CCA mode, which can be controlled based on various fabrication parameters^25,27^. However, both SLIPSs and SOCAL surfaces as well as other coating procedures require a multiple stage chemistry process with tight control of the manufacturing parameters required to achieve the lowest hysteresis.

One-step surface functionalisation via physical or chemical modification and/or the application of coatings, have also been developed. Physical surface modification methods include laser etching, electrospinning, and solvent processing, amongst others. These methods are generally well established and commercially available, but introduce complications arising from the additional roughness imposed or from surface contamination^42^. While the use of chemical modification methods, such as Chemical Vapour Deposition (CVD), can eliminate this issue as well as increase durability. Nonetheless, the need for an environmental chamber and vacuum for the fabrication of high-quality coatings limits CVD’s widespread practicability. In addition to CVD, spray-coating, dip-coating, and spin-coating, can also be defined as surface coating methods which have basic and scalable processing^43–45^. Amongst these methods, polymer grafting represents an attractive additional surface modification technique rendering an intrinsically hydrophilic surface into a hydrophobic one with ${\theta }_{{\text{e}}}$ = 97° ± 10° and CAH of 20°^34^.

More recently, the optimisation of the different grafting parameters such as oil viscosity, volume, method of deposition, as well as the number of layers, allowed for the fabrication of hydrophobic surfaces with ${\theta }_{{\text{e}}}$ ~ 108° and CAH as low as 1°^46^. In addition, the investigated parameters allowed for the tuning of the droplet-surface interactions playing a paramount role on the CAH with values ranging between as low as 1° to 20°, which anticipates different evaporation dynamics^46^. Besides the CCR and CCA evaporation modes introduced above, often, on non-ideal surfaces, droplets evaporate in the mixed mode where both the contact angle and radius decrease with time, which typically ensues at the end of the evaporation^47,48^.

Here we report on the evaporation behaviour of pure water droplets on simple to fabricate and scalable silicone oil grafted surfaces varying in the fabrication method and hence in the contact angle hysteresis paying special attention to the dynamics of the contact line. Overall, the droplet-surface interactions as well as the pinning forces influencing the motion of the contact line are thus reduced along with the contact angle hysteresis when compared to the smooth hydrophilic silicon original surface. In addition, we use Teflon coated silicon wafer to benchmark our silicone oil grafting procedure to a well-stablished hydrophobic coating. More specifically, the duration of the different evaporative behaviours dictated by the droplet-surface intimate interactions and pinning forces can be then tuned between those reported on silicon and on Teflon depending on the fabrication parameters chosen. This work provides the relevant guidelines for controlling the evaporation behaviour and motion of the contact line based on the grafting parameters chosen, which may find usefulness in many industrial and everyday applications.

## Methods

### Surfaces preparation

The base substrate is bare silicon wafer, hydrophilic in nature, diced in 1 cm × 1 cm sample size. Bare silicon is also used as the hydrophilic substrate as received, while Teflon coated silicon is utilised as the hydrophobic substrate. The Teflon coated substrates are fabricated following the procedure reported in Orejon et al.^20^. as follows: a Teflon layer of 1 µm thickness is spin coated on a silicon wafer, followed by annealing at 330 °C in a furnace and then cleaning in an ultrasonic bath of isopropanol for 15 min.

Whereas the silicone oil grafted samples are prepared following the work of Eifert et al.^34^ while supressing the PDMS brushes washing step and further impregnation step reported earlier while still achieving slippery water repellent low hysteresis oil grafted surfaces as reported in Abbas et al.^46^. Firstly, the substrates are cleaned in an ultrasonic bath (Fisher Scientific UK Ltd, FB15047) with pure acetone and then pure ethanol (Sigma Aldrich) for 10 min respectively, followed by rinsing with de-ionised water and drying with filtered compressed air. Subsequently, to ensure that the surface is completely free of contaminants including any volatile organic compounds^49^, the samples are placed into a Henniker Plasma cleaner (HPT-200) at 60 W (30% power) and 15 sccm (standard cubic centimetre per minute) for 5 min. Thereafter silicone oil (Sigma Aldrich) varying in viscosity is applied to the different plasma cleaned bare silicon surfaces via two different methods; the pipette and the dip-coating methods, before subjecting them to high temperature grafting. The pipette method is used to apply either 2 µL, 5 µL, or 10 µL of low and medium viscosity oils (5 and 20 cSt respectively) for each grafted layer, while the dip-coating method with different speeds is used to apply the different layers for medium and high viscosity oils of 20 cSt and 100 cSt. The dip-coating method is implemented for medium and high viscosity oil so to apply a more homogeneous layer of oil on surface. For the dip-coating method, samples are attached with a glass slide using double-sided tape and immersed in the silicone oil bath containing the relevant oil using a dip-coater (Ossila). To compare the effect of oil application method, 5 cSt oil samples are also prepared via dip-coating. For 5 cSt, 20 cSt oil and 100 cSt oils, the dip-coater speed is set at 0.2 mm/s, 0.05 mm/s and 0.01 mm/s respectively.

The samples are then placed on a heating plate (Fisher Scientific UK Ltd) at temperatures ranging from 200 to 250 °C allowing sufficient time for the oil to covalently bond to the surface, forming a thin layer of PDMS chains after evaporation. The oils showed preferential evaporation when applied either via the pipette or subjected to high grafting temperatures (300 °C) for high viscosity oils; hence, the dip coating method only and the lowest of the temperatures are used for 100 cSt high viscosity oil. Grafting procedure was carried out for each of the layers applied and the number of grafted layers ranged between 1 and 5. While no PDMS brushes rinsing step was involved after the grafting procedure and compressed air was used to remove any excess of dust and potentially that of oil, it is necessary to acknowledge that some amount of oil may still remain on the surface even after the high temperature grafting procedure applied in this work. Nonetheless, experimental observations of the droplet profile evaporation in time presented in Fig. 5a confirm the absence of a visible wetting ridge while the low thickness of the coatings within hundreds of nanometres reported in Table 2 additionally supports the absence of excess oil.

Table 1 summarises the fabrication parameters as well as the corresponding CA and CAH values of all prepared samples. CA and CAH values can also be retrieved from Abbas et al. in Ref.^46^. For the dip-coating method, the Landau-Levich-Derjaguin (LLD) equation is used to estimate the thickness of the deposited oil^50^, which is then multiplied by the substrate area to calculate the volume deposited as reported in Table 1. We note here that the volume of the oil is to some extent related to the amount of silicone oil grafted and/or remaining oil present on the surface as well as to the thickness and the roughness of the coating as it will be discussed in the Coating thickness and roughness subsection.

**Table 1:**
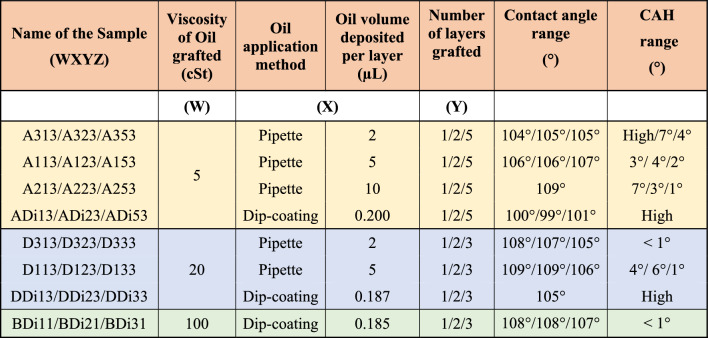
Fabrication parameters for grafting silicone oil on silicon substrates and corresponding CA and CAH values^46^.

Name of the sample as WXYZ where W is the viscosity of the oil 5 cSt (A), 20 cSt (D), and 100 cSt (B), X is the application method as volume for the pipette method volumes of 2 µL (3), 5 µL (1), and 10 µL (2), or dip coating (Di), Y is the number of layers and Z is the grafting temperature either as 250 °C (Z = 3) or 200 °C (Z = 1). *High* contact angle hysteresis (CAH) range indicates complete pinning of the contact line.

### Experimental setup and procedure

**Figure 1:**
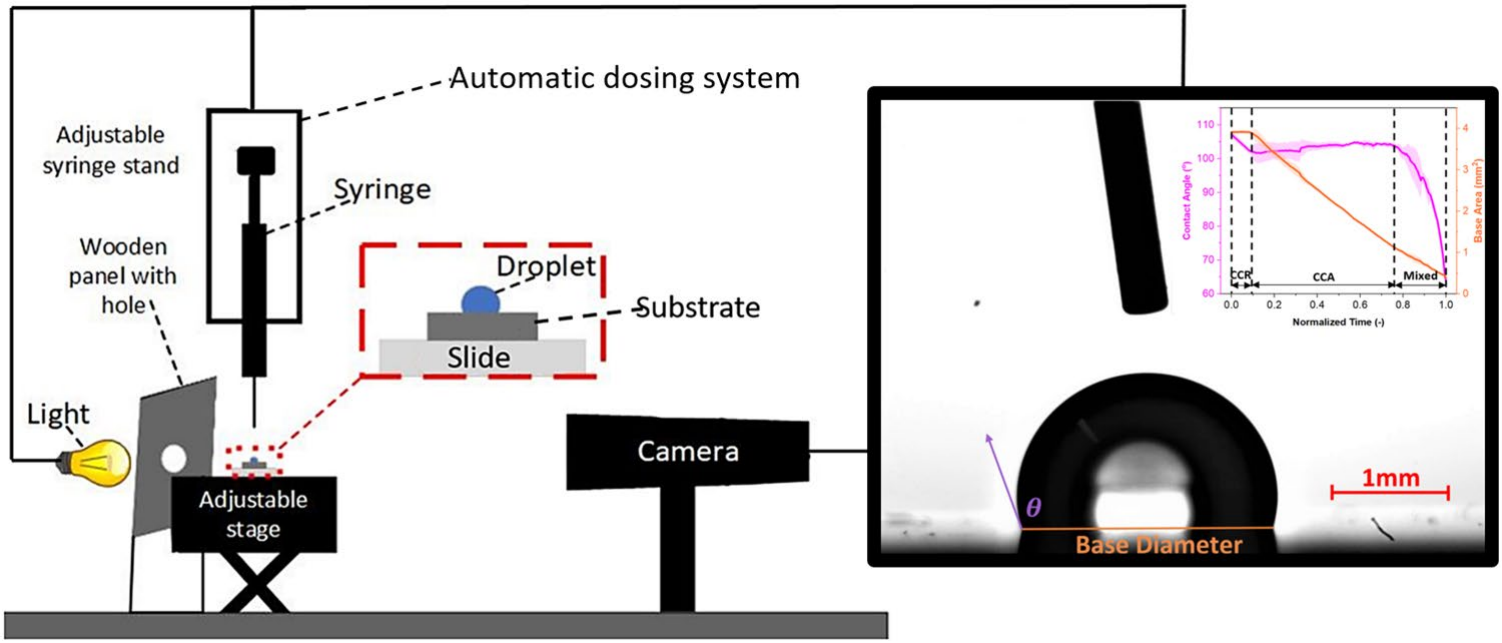
Schematic diagram of custom-built drop shape analyzer (DSA) used for droplet evaporation experiments. It consists of an adjustable *x*–*y*–*z* stage to place the samples, a light source with a wooden panel in front of it to focus the light on the sample through a hole in it, an automated dosing system containing a syringe and a needle, and a camera to record the droplet evaporation videos. A typical snapshot of the observations can be found as inset where the scale bar is 1 mm. Solid lines represent the different electronic connections between the PC and the camera, the syringe pump and the back light.

The droplet evaporation dynamics of de**-**ionised water (surface tension: 72 mN/m at ambient temperature and pressure) on the fabricated substrates are investigated using a custom-build Drop Shape Analyzer (DSA), whose schematic is shown in Fig. 1. It consists of an automated dosing system (Cellix, Exigo) including syringe and needle, a camera (IDS, UI-3880CP-C-HQ), a back light (Viltrox, L116T) and a manually controlled *x*–*y*–*z* stage (Thorlabs, PT3/M). The dosing system is used to place a small water droplet of controlled volume 4 ± 0.5 µL on the samples, i.e., droplet sizes below the capillary length for water of approximately 2.7 mm at ambient conditions. Compressed air is used to remove any dust particles that may have settled on the coating surface prior to evaporation experiments. A video is recorded at the rate of one frame per second, until the droplet has reduced from a volume of 4 µL to approximately 0.05 µL, which is this this latter the limit for the accurate processing of the droplet images. To understand the droplet shape evolution and dynamics of the triple-phase contact line during evaporation, the values of the CA and droplet radius are extracted from the recorded videos using an in-house Python program, pyDSA^51^. To ensure continuity, the volume is calculated by making use of the CA and the droplet radius assuming the droplet as a spherical cap with a circular footprint, which is confirmed from experimental observations where the droplet profile exhibits circular arcs consistent with spherical caps and by the rather mobile contact line. All experiments were carried out under ambient room conditions, which are at ambient temperature of 21  ± 3 °C, relative humidity of 40 ± 10% and ambient pressure of 1 atm. All experiments are repeated at least three times and the average as a solid line as well as standard deviation as shaded area are reported for each case. For analysis, the CA and the base area are plotted against normalised time. From the droplet profiles, the time spent in the CCR, CCA and mixed modes is then obtained for each run for droplets evaporating on each substrate. We note here that during the reported CCA mode of evaporation, there is some finite occurrence of stick–slip behaviour on all surfaces (except 1 layer 100 cSt oil grafted). Though for simplicity, since the CA remains in a similar range during evaporation, we group such evaporation regime within the CCA mode firstly, while further analysis on the frequency and strength/magnitude of the stick–slip occurrence is also carried out. Finally, to obtain the data representing the stick–slip behaviour of the contact line, the length that the droplets base diameter retracted is then extracted from the droplet profiles and the number of times this length is within the stick–slip range is further computed. The 4 ranges of stick-slips grouped are; between 0.015 and 0.025 mm, 0.025 mm and 0.035 mm, 0.035 mm and 0.045 mm, and greater than 0.045 mm. Last, changes in contact angle and magnitude of the contact line jumps are then used to calculate the average pinning force on the different substrates function of the fabrication procedure adopted.

### Atomic force microscope (AFM) surface characterisation

A NanoWizard^®^ V AFM from JPK Instruments and an uncoated silicon non-contact PointProbe^®^ Plus in soft tapping mode is used to measure the PDMS root mean square (RMS) roughness, *R*_*rms*_. While an uncoated silicon cantilever from NANOSENSORS™ with an applied force of 30 nN is utilised in the force-mapping mode for the thickness measurements. Force-mapping mode is used to record the Force-Distance (FD) curves at three different locations on four representative samples, where the tip of the cantilever approaches and retracts repeatedly on a 1 µm × 1 µm area (16 × 16 points grid) each time. These FD curves are later analysed to extract the thickness of the coatings.

## Results and discussion

This section introduces and explains the diversity of droplet evaporation behaviours or modes and their durations that can be tuned by altering the various surface fabrication parameters introduced in the Methods Section. First, the droplet evaporation behaviour on bare hydrophilic silicon and hydrophobic Teflon as benchmarked surfaces is presented, analysed and described. This is followed by the results, analysis and discussion on the silicone oil grafted surfaces reported here in terms of evaporation modes and their durations as well as the magnitudes and frequency of the stick–slip events and the pinning/de-pinning forces involved during the evaporation function of the fabrication parameters. The various fabrication parameters investigated include viscosity, volume, oil deposition method as well as the number of layers of oil grafted.

### Droplet evaporation on bare silicon and Teflon

**Figure 2:**
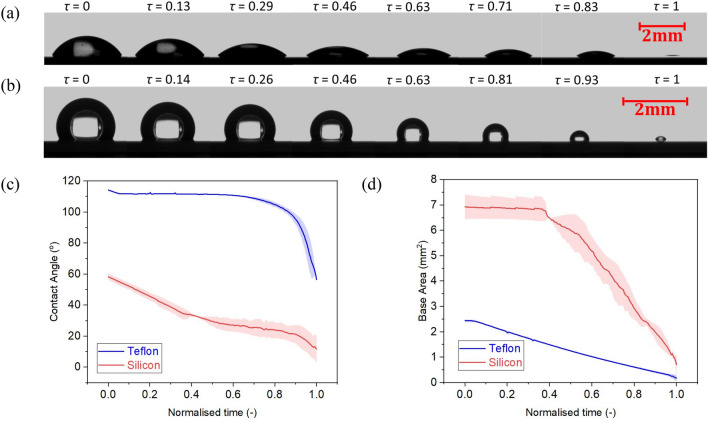
Droplet evaporation snapshots at different intervals of time represented as normalised time *τ* (–) on **(a)** bare hydrophilic silicon and **(b)** hydrophobic Teflon. **(c)** Contact angle (°) and **(d)** droplet base area (mm^2^) variation versus normalised time *τ* (–) during evaporation on bare silicon and on Teflon^20^. Solid lines represent the average and shaded areas represent the standard deviations for at least 2 independent experiments.

The evaporation behaviour as contact angle and as base area function of normalised time for a pure water droplet on an intrinsically hydrophilic bare silicon surface and on a hydrophobic Teflon coated surface are represented in Fig. 2a, b. Normalised time is calculated by dividing the time at a given instant by the total droplet evaporation time. Note that normalised time is used to represent the evaporation behaviour since the total evaporation time on such surfaces may differ by two to threefold. The different evaporation time depends whether a droplet evaporates in the CCR or CCA modes, on the substrate thermal conductivity, ambient humidity, amongst others, as earlier reported in the literature^8,20,52,53^.

To highlight is the two very different evaporation patterns function of the surface wettability on bare silicon and Teflon represented in Fig. 2. A sessile water droplet on hydrophilic bare silicon surfaces displays an initial contact angle ~ 60°, which is owed to the hydrophilicity of the substrate. Then, in a water vapour unsaturated environment, after deposition, the droplet undergoes evaporation firstly in the CCR mode for approximately 40% of the droplet lifetime, and then transitions into the CCA mode for a further 50% of its lifetime. The droplets on bare silicon substrates vanish in the mixed mode for the last 10% of the droplet lifetime, where both the contact line and the contact angle decrease in time. A sessile water droplet on a hydrophobic Teflon substrate displays a higher initial contact angle of ~ 114°. On a smooth hydrophobic surface, evaporation typically initiates in the CCR mode with a pinned contact line for only the first 4% of the total evaporation time. Thereafter, it is more favourable for the base area to reduce linearly, while the contact angle remains constant, hence evaporation ensues following the CCA mode for the next 79% of the total evaporation time^20,54^. Last, after the CCA mode, the droplet evaporates in the mixed mode for the remaining 17% of the evaporation lifetime. The high initial contact angle coupled to the short duration of the CCR mode is attributed to the low surface energy and lack of physical and chemical heterogeneities of the benchmarked Teflon coated surface. Despite the root mean square roughness (*R*_*rms*_) of plain silicone, obtained by Atomic Force Microscopy (AFM), being sevenfold smaller than that of Teflon, for 0.12 nm and 0.88 nm respectively^20^, larger initial pinning and absence of CCA mode of evaporation is reported on the silicon surface, which is then attributed to its intrinsic hydrophilic wettability. Once, we have established differences in both the surface wettability and the water droplet evaporation behaviour between the benchmarked opposing wettability, hydrophilic and hydrophobic, surfaces and their agreement with the literature^20^, next we introduce and discuss the different evaporation behaviours on our silicone oil grafted samples function of the fabrication parameters.

Besides the three classic main evaporation regimes reported to date, which are the CCR, CCA and mixed modes, many studies have reported an additional stick–slip mode of evaporation. Stick–slip evaporation mode is characterised by discrete contact line pinning events while the contact angle decreases to account for the fluid evaporated followed by sudden decrease in the contact radius coupled with the increase in contact angle during a de-pinning event.^20^. In the stick–slip mode, during a de-pinning event the rapid receding/slip contact line motion occurs with the contact angle regaining an earlier similar initial value^20^. Hence, for simplicity observations reporting some finite stick–slip behaviour with the contact angle oscillating within certain values was grouped within the CCA evaporation mode firstly, while further analysis on the stick–slip occurrence is provided thereafter.

### Effect of grafted oil volume

**Figure 3:**
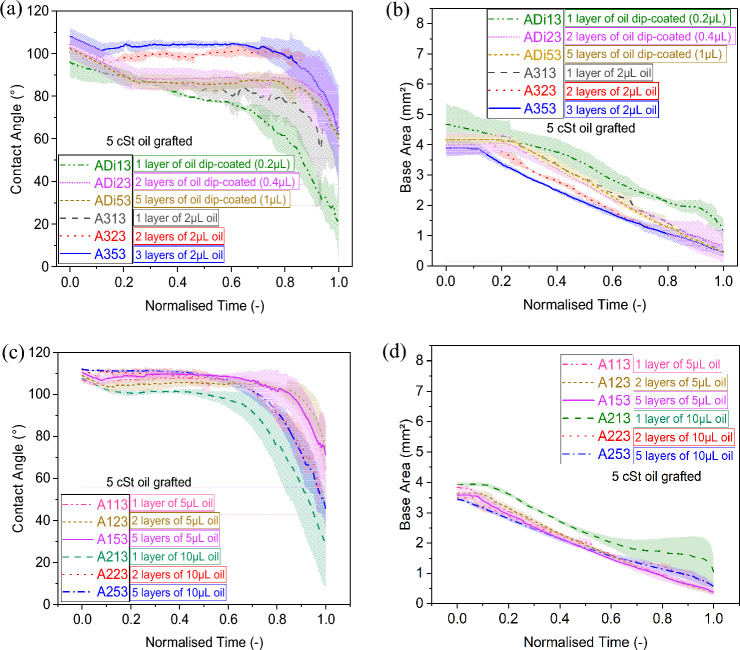
**(a,c)** Contact angle (°) and **(b,d)** base area (mm^2^) function of normalised time (–) for a 4 µL water droplet evaporation on 5 cSt silicone oil grafted samples (A-samples). **(a,b)** low volumes of oil grafted (0.2 µL via the dip-coating method per layer and 2 µL via the pipette method per layer) and **(c,d)** high volumes of oil grafted (5 µL via the pipette method per layer and 10 µL via the pipette method per layer). Solid, dashed, dotted and dashed-dotted lines represents the average and shaded areas the standard deviations for at least 3 independent droplet evaporation experiments for each of the surface grafting conditions represented. Refer to Table 1 for the different details on the substrate nomenclature.

Next, we compare the effect of the silicone oil grafted volume on the evaporation behaviour of pure water droplets. Figure 3 displays the contact angle and base area function of normalised time for droplets evaporating on substrates grafted with 5 cSt silicone oil following varying volumes and deposition methods. On one hand, 2 µL, 5 µL, and 10 µL silicone oil volume is applied per layer on cleaned samples via the pipette method earlier described in the Methods Section. Whereas on the other hand, the dip-coating method ensures the accurate and uniform thin deposition of ~ 0.2 µL of silicone oil for each layer according to the Landau-Levich-Derjaguin LLD equation^55^ as Eq. (2) below;

2 $$h=0.946 \sqrt{\frac{\gamma }{\rho g}}{Ca}^\frac{2}{3}$$

where *γ* is the surface tension of the oil, *ρ* is the density of the oil, *g* is gravitational acceleration, and *Ca* is the capillary number. For simplicity in the representation and comparison, we group the results for 0.2 µL via dip-coating and 2 µL via the pipette method in Fig. 3a, b; while the results for 5 µL, and 10 µL via the pipette method are found in Fig. 3c, d.

When comparing Fig. 3a,c the initial contact angle of the droplets ranges from ~ 95° to ~ 109°. A greater initial contact angle is observed in the case of high volume of oil grafted such as sample A353 when compared to low volume of oil grafted sample ADi13. When looking into the contact angle and contact radius profiles, these resemble qualitatively each other in terms of overall evaporation behaviour. To highlight is the qualitative resemblance of the evaporation behaviour, independently of the surface grafting parameters, somewhere in between the evaporation behaviours on Teflon and on silicon earlier reported in Fig. 2, which will be further qualitatively and quantitatively compared in the Discussion and Analysis section. In particular, the greater the volume of silicone oil grafted, the closer the evaporation behaviour is to that of the benchmarked hydrophobic Teflon surface, while the least the volume grafted, the closer the evaporation behaviour is to that of benchmarked hydrophilic silicon surface. In the case of one layer coated samples, the degree/time of initial pinning of the contact line decreases as the volume of oil used per layer increases up to 5 µL per layer and then increases for 10 µL per layer. Such decrease and then increase in pinning time related to hysteresis resembles the CAH function of the PDMS molecular weight trend reported recently in the review by Chen et al.^33^, except for the 1-layer dip-coated surface where there seems to be absence of strong contact line pinning. In the case of drop coated samples, the greater volume of grafted silicone oil can reduce the initial pinning time of the droplet lifetime from 11% to 26% (Fig. 3b) down to 1%–14% (Fig. 3d). The rather larger duration of the CCR mode on low volume grafted samples, when compared to benchmarked Teflon (Fig. 2), can be attributed to the absence and non-uniformity of the grafted silicone oil coating due to insufficient amount of oil applied (Fig. 3a, b). After the first pinning stage, the contact line then monotonically recedes while the contact angle remains constant and/or oscillating around similar values in the CCA mode during most of the droplet lifetime. Last, evaporation ensues in the mixed mode for the last 80% to 99% of the droplet lifetime for low grafted volumes (Fig. 3a, b) and for the last 65% to 99% of the droplet lifetime for high grafted volumes (Fig. 3c, d). From the evaporation behaviours reported here, we can conclude that grafting more volume ensures the less rough and the more uniform coating coverage of the substrate as later shown in Fig. 7, which in turn minimises the likelihood of pinning sites and predominance of the observed CCA mode with shorter initial pinning times as reported in Chen *et* *al.*^33^. We also highlight here the reproducibility and alike of the evaporation behaviour for 5 µL or 10 µL of silicone oil grafted samples independently of the number of layers deposited with initial pinning times smaller than 15% of the total evaporation time and initial contact angles ranging between 107° and 112°. The different durations of the evaporation regimes are then summarised and compared for all the different grafting fabrication parameters investigated as well as those in the literature withtin the Discussion and Analysis section.

### Effect of grafted oil number of layers

**Figure 4:**
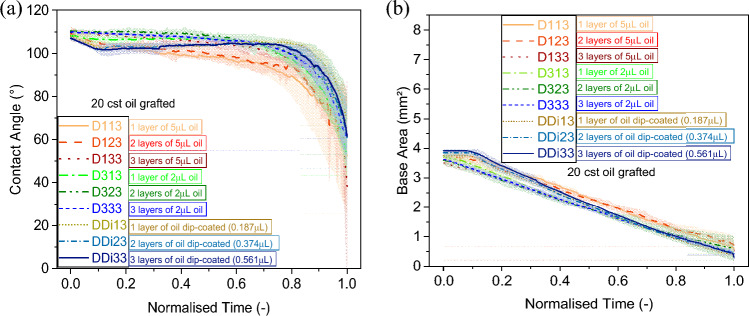
**(a)** Contact angle (°) and **(b)** base area (mm^2^) function of normalised time (–) for a 4 µL water droplet evaporation on 20 cSt silicone oil grafted samples D-Samples with 1, 2 and 3 layers fabricated by deposition of 0.187 µL per layer via the dip-coating method, and the pipette method for 2 µL and for 5 µL per layer. Solid, dashed, dotted, dashed-dotted and dashed-dotted-dotted lines represent the average and shaded areas represents the standard deviations for at least 3 independent droplet evaporation experiments for each of the surface grafting conditions.

**Figure 5:**
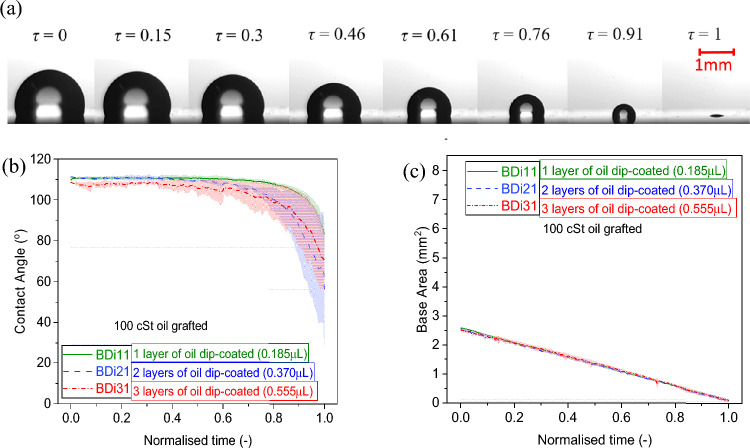
**(a)** Droplet evaporation snapshots at different intervals of time represented as normalised time *τ* (–) on 3 layers of 100 cSt silicone oil grafted sample prepared by the dip-coating method: BDi31. **(b)** Contact angle (°) and **(c)** base area (mm^2^) function of normalised time (–) 4 µL water droplet evaporation on 100 cSt silicone oil grafted samples of 1, 2 and 3 layers by deposition of 0.185 µL per layer via the dip-coating method. Solid, dashed and dashed-dotted lines represent the average and shaded areas represent the standard deviations for at least 3 independent droplet evaporation experiments for each of the surface grafting conditions.

This section provides insights on the effect of number of grafted layers on the droplet evaporation behaviour. The different number of layers were applied as: 1, 2 and 5 layers for 5 cSt and 1, 2 and 3 layers for 20 cSt and for 100 cSt grafted silicone oil. Within Fig. 4, we make use of the evaporation results for 20 cSt grafted silicone oil following the different deposition methods, different volumes and various number of layers. In particular, 20 cSt oil samples are prepared by applying 0.187 µL (per layer) volume using the dip-coating method and 2, 5 µL volumes (per layer) using the pipette method with the number of layers ranging from 1 to 3 instead. For 20 cSt oil, we do not consider 10 µL oil grafting or higher number of layers since under those fabrication conditions, preferential evaporation of the silicone oil is observed, which resulted in rather heterogeneous grafting of the surface. We also note here that within this subsection, we refrain from referring to 100 cSt results since the number of layers applied did not induce any obvious change in either the wettability and/or the evaporation behaviour as it will be conveyed within the next subsection and in Fig. 5. Hence, Fig. 4 focuses on the effect of number of layers on medium viscosity silicone oil 20 cSt. We note here that this subsection also refers to the results presented in Fig. 3, where the evaporation behaviour on 5 cSt silicone oil grafted samples for different number of layers ranging from 1 to 5 layers and prepared by applying 0.2 µL (per layer) volume via dip-coating and 2, 5 and 10 µL volumes (per layer) via pipette method, were reported.

When looking into 5 cSt grafted silicone oil substrates, the initial contact angle represented in Fig. 3a increases with the number of layers on samples grafted with low volumes per layer (0.2 µL and 2 µL). On the other hand, on substrates where high volumes of 5 cSt silicone oil have been grafted (5 µL and 10 µL), an increase in the initial contact angle is observed when considering 2 layers instead of 1; however, no additional changes are observed when adding more than 2 layers as shown in Fig. 3c. The number of grafted layers has a similar effect to that of the volume of silicone oil grafted where the use of small volumes, especially in case of low viscosity (hence lower molecular weight) silicone oil, can lead to short PDMS chains grafting with some uncoated patches^56^, which promotes droplet pinning. While as the number of layers increases, the silicone oil grafted is distributed more uniformly fully covering the surface with the observed decrease in the CAH^46^ and in the initial pinning stage duration as clearly conveyed in earlier subsection.

When looking at the substrates grafted with 20 cSt silicone oil reported in Fig. 4, the number of layers have a negligible impact on the initial contact angle, represented in Fig. 4a, with all values ranging between 106° and 111° independently of the amount of oil deposited via either the pipette or dip-coating method. Nonetheless, when focusing on the evaporation behaviour and the dynamics of the contact line represented in Fig. 4b, the initial CCR mode duration decreases from 13%  to 1% with the increase of the number of layers for 5 µL volume grafted via the pipette method. Whereas in the case of the dip-coated method and 2 µL volume grafted, the number of layers seems to not have a major impact on the duration of the CCR mode, which is approximately 1% or less for all 2 µL volume of oil grafted and 10% for all dip-coated layers. Although the number of layers has no major effect on the duration of the initial pinning/CCR and CCA modes for low volumes of 20 cSt silicone oil deposited via the dip-coating method; nonetheless, increasing the number of layers from 1 to 3 plays a major role on the duration of the evaporation modes when grafting higher volumes using the pipette method.

From both Figs. 3 and 4, for the pipette method, the lower the number of layers the larger the standard deviations in the evaporation data whereas for the dip coating method there are not large differences in the standard deviations reported when looking at the number of layers. This trend can presumably be attributed to the incomplete or less homogeneous surface coverage of the grafted oil when applying low volumes of low and medium viscosity oils via the pipette method. The above clearly points out the importance of subsequent grafting since the first PDMS grafted layer attaches to the bare silicon substrate while subsequent silicone oil grafted layers may attach to already grafted PDMS layers or cover earlier empty areas thus creating a more homogenous and more dense silicone oil coating^57^.

### Effect of grafted oil viscosity

Next, we look into the effect of silicone oil viscosity by comparing the results for 5 cSt oil (both dip coating and pipette methods) represented in Fig. 3, for 20 cSt oil (both dip coating and pipette methods) shown in Fig. 4, and for 100 cSt oil (dip coating method) included in Fig. 5.

Snapshots of a droplet evaporation on 1 layer 100 cSt silicone oil grafted sample BDi11 at different intervals of time exemplifying the continuous receding motion of the contact line during most of the evaporation lifetime are represented in Fig. 5a, while the respective contact angle and base area evaporation profiles function of normalised time are represented in Fig. 5b and Fig. 5c respectively. The initial contact angle of a water droplet on 20 cSt oil (Fig. 4a) and 100 cSt (Fig. 5b) silicone oil grafted surfaces making use of low volumes of the grafted oil via dip-coating is ~ 5° to 9° higher than for 5 cSt (Fig. 3a, c). When looking into the evaporation behaviour it is clear that the greater the oil viscosity, the lower the droplet pinning with the consequent shorter duration of the CCR mode. The CCR mode lasts for 1% or less on 100 cSt oil grafted samples (Fig. 5c), while it ranges between 1% and 13% for 20 cSt and between 1% and 26% for 5 cSt, i.e., increases with decreasing the viscosity of the oil. Thereafter, depending on the fabrication procedure, evaporation ensues in either the CCA mode/stick–slip mode for 50% to 75% of the droplet lifetime and/or in the mixed mode for 13% to 35% for 5 cSt, 18% to 34% for 20 cSt and 28% to 48% for 100 cSt. These variations in the durations of the different evaporation modes are attributed to the different degree and homogeneity of the grafted silicone oil altering the surface physical chemistry, which is function of the viscosity, the volume, the deposition method, and the layers of the oil grafted^33,58,59^. The absence of contact line pinning and occurrence of CCA mode and/or the stick–slip mode during most of the droplet evaporation on surfaces grafted with 100 cSt high viscosity oils are further supported by their low CAH and low sliding angles earlier reported in the literature^46^, which anticipated a very mobile contact line during evaporation. Further, the results and behaviour presented here agree well with those reported on PDMS films with an intermediate curing ratio of 40:1 reported by Chen et al., where water droplets show approximately no CCR mode at the beginning of the evaporation while thereafter most of the evaporation, i.e., ~ 72% of the droplet lifetime, is spent in the CCA mode, and then followed by the mixed mode^60^.

To be able minimise evaporation in the CCR mode and maximise that on the CCA mode for substrates grafted with 5 cSt low viscosity silicone oil, the grafting of multiple layers of high volumes (10 µL) via the pipette method is recommended. While for lower volumes and lesser number of layers, CCR and stick–slip behaviours are the main evaporation mechanisms ensuing. On the other hand, for 100 cSt high viscosity oil, only the deposition of a single layer of oil via the dip-coating method is required so to minimise the CCR mode down to < 1%. Despites the rather smooth receding motion of the contact line and the similar behaviour of droplets evaporating on 100 cSt high viscosity oil grafted substrates when compared to benchmarked Teflon, we must note here that there is still small discrete pinning and depinning events in the form of stick–slip taking place except for BDi11. Stick–slip behaviour increases in frequency/occurrences with number of layers (from 0 to 15 for 0.015 < δ*R* < 0.025 and from 0 to 4 for δ*R* < 0.045 ranges). This is further presented and discussed in detail in the Discussion and Analysis section.

### Coating thickness and roughness

Next, we present the AFM results and analysis of surface roughness and coating thickness on four representative samples: A113, A153, BDi11 and BDi31. The choice of the samples was their contrasting evaporation behavior where A113 evaporates in the CCR mode for most of the droplet lifetime while BDi11 suppresses the CCR mode and evaporates mainly in the CCA mode and then in the mixed mode. These samples also allow to look at the effect of grafted layers numbers on the surface roughness and coating thickness.  

**Figure 6:**
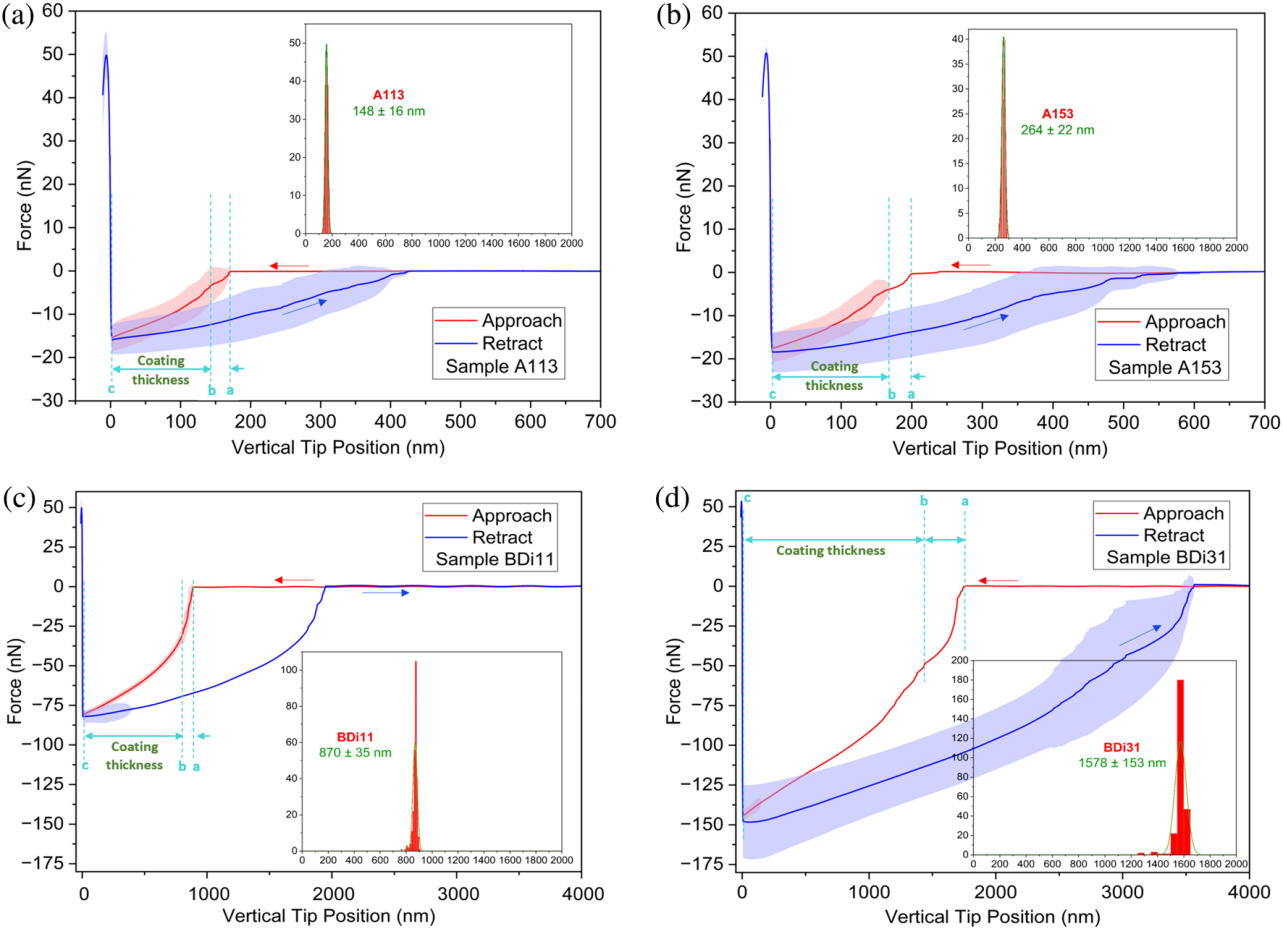
Representative AFM Force-Distance (FD) curves for four representative samples **(a)** A113 (one layer 5 cSt grafted). **(b)** A153 (five layers 5 cSt grafted). **(c)** BDi11 (one layer 100 cSt grafted). **(d**) BDi31 (three layers 100 cSt grafted). A 30 nN applied force is used for each scan on 1 µm × 1 µm area (16 × 16 point grid). Shaded area is used to represent the standard deviation of up to 30 curves (10 from each scan). Insets (histograms) show the average PDMS coating thickness of 768 curves (256 for each region).

**Figure 7:**
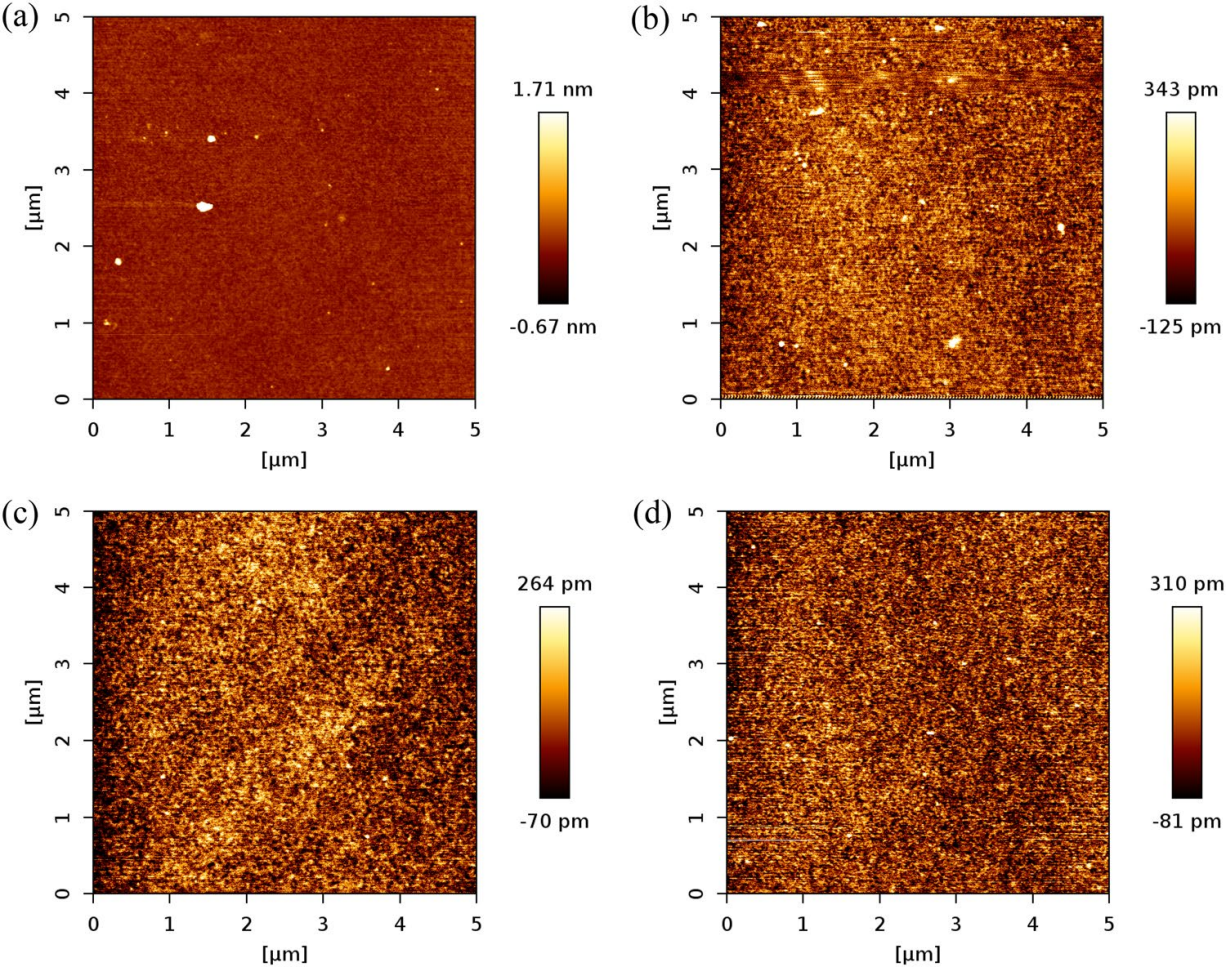
Representative AFM scans showing the surface topgraphy profiles for samples. **(a)** A113 (one layer 5 cSt grafted). **(b)** A153 (five layers 5 cSt grafted). **(c)** BDi11 (one layer 100 cSt grafted). **(d**) BDi31 (three layers 100 cSt grafted). The scans are performed in the soft taping mode on an area of 5 µm × 5 µm.

On one hand, in order to retrieve the thickness of the grafted layer/s Fig. 6 presents AFM Force-Distance (FD) curves in the force-mapping mode in terms of force (nN) and tip position (nm) during approaching and retraction on three different regions of the sample. The AFM Force-Distance (FD) curves include the average and standard deviation of ten curves from each scan region, i.e., a total of 30 representative curves. From the force to vertical tip position curves, a sudden negative force as the tip approaches the surface is observed, which is attributed to capillary forces interacting with the tip. This sudden dip distance, which is depicted by the vertical blue dashed lines a and b, increases as the viscosity of the oil grafted does, which suggests the presence of stronger capillary forces for the high viscosity grafted oil. Thereafter the force to tip position decreases nearly linearly between b and c, vertical blue dashed lines indicating the motion of the tip through the grafted layer/s. This behaviour is characteristic of PDMS coatings^56,61–63^ and the approach to estimate the thickness of the coating from the force to tip distance data was adopted from the work of Teisala et al.^56^ where the distance b to c in Fig. 6 corresponds to the coating thickness. The inset histograms in Fig. 6 provide the average of the coating thickness for all the 768 curves, i.e., 256 scan per region, while the average values and standard deviation of the coating thickness have been further included in Table 2. On the other hand, Fig. 7 includes AFM surface topography profile measurements performed in the soft tapping mode to an area of 5 µm by 5 µm from which the root mean square (RMS) roughness, *R*_*rms*_, is calculated.

**Table 2:**
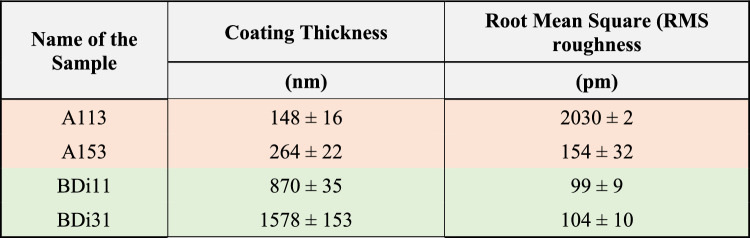
Coating thickness and root mean square (RMS) roughness, *R*_*rms*_, for four representative sample, calculated using the representative AFM Force-Distance (FD) curves and AFM surface topography profiles reported in Fig. 6 and Fig. 7 respectively.

While the summary of the coating thickness and root mean square roughness are additionally provided in Table 2.

When looking into Figs. 6, 7 and Table 2, 5 cSt oil grafted surfaces present the lowest coating thicknesses, which are equal to 148 ± 16 nm for one layer grafted sample A113 and increases to 264 ± 22 nm as the number of grafted layers increases from one to five in the case of A153. As more layers are grafted on top of each other the thickness of the coating increases while at the same time the surface becomes smoother as the surface topography profile ranges decrease, which is evident when comparing Fig. 7a, Fig. 7b and the results presented in Table 2. This is owed to the more homogeneous surface coverage of the coating as subsequent grafted layers are implemented. The better surface coverage of the coating and the smoother of the profiles empower the more prolonged droplet evaporation in the CCA mode when compare to the one layer grafted surface as earlier shown in Fig. 3.

When looking into the 100 cSt oil grafted samples, greater thickness of the coatings are expected as per the greater viscosity, longer chains and higher molecular weight of the grafted oil, which is confirmed by the reported values in Table 2 as 870 ± 35 nm for one layer BDi11 sample and 1578 ± 153 nm for BDi31 sample after grafting two more layers on top of first one. In the case of high viscosity oil 100 cSt, although the coating thickness increases it is worth noting that the surface is rather smooth and there is no change in the RMS roughness, *R*_*rms*_, with values in the order of 100 pm or 0.1 nm as demonstrated when comparing Fig. 7c, d. The very small volume of oil applied uniformly via dip-coating (Table 1) results in a rather homogenous layer even after grafting 3 layers. The smoothness of the 100 cSt oil grafted samples with values eightfold smaller than those reported on Teflon^20^ supports the absence of initial pinning and absence of stick–slip phenomenon with larger duration of the CCA mode during evaporation.

It is worth stating here that although there might be some small amount of ungrafted oil residue present due to the suppression of the rinsing step, Gresham et al. observed no difference on the CAH prior and after extensive washing steps (in toluene, acetone and water and/or via CO_2_ snow jet cleaning) for 20 and 50 cSt grafted surfaces with the lowest CAH being around 1.5°^64^. They concluded that the slippery behaviour of the prepared surfaces was not due to the presence of remaining unreacted PDMS oil. Moreover, experimental results reported in Abbas *et al*. for 100 cSt grafted surface immersed in ethanol for 7 h also did not show major apparent changes in either the CA or the CAH prior and after immersion^46^. Furthermore, Krumpfer & McCarthy^65^ also did not observed major differences in the CAH prior and after rinsing in toluene, acetone and water for 20 cSt and 200 cSt grafted silicone oil on silicon wafers, which are comparable to the values reported in Table 1. Hence, additional rinsing step should not affect the results in terms of wettability and evaporation behaviour.

Nonetheless, since the PDMS brushes step and impregnation steps earlier reported in the work of Eifert et al. were suppressed to achieve our slippery water repellent low hysteresis oil grafted surfaces^34^, we acknowledge the potential presence of oil after the high temperature grafting procedure carried out here. This is also additionally suggested by the greater thicknesses of the grafted samples presented in this work of at least one or two orders of magnitude greater when compared to those of Eifert et al. and Teisala et al.^34,56^. And by the sudden negative force as the tip approaches the surface as reported in Fig. 6, which is attributed to capillary forces interacting with the tip and increases increases as the number of layers and as the viscosity of the oil increases.

## Discussion and analysis

Next, we make use of only five representative samples so to provide a direct comparison between the different evaporation behaviours on the different substrates prepared by varying the different fabrication parameters, for simplicity. In this section, the effect of oil viscosity, deposition method, volume deposited, and number of layers, on the evaporation modes and their durations, on the magnitude and frequency of the stick–slip jumps, and on the pinning/de-pinning forces involved, is quantified and compared for these five representative samples along with the benchmarked results for bare silicon and Teflon.

**Figure 8:**
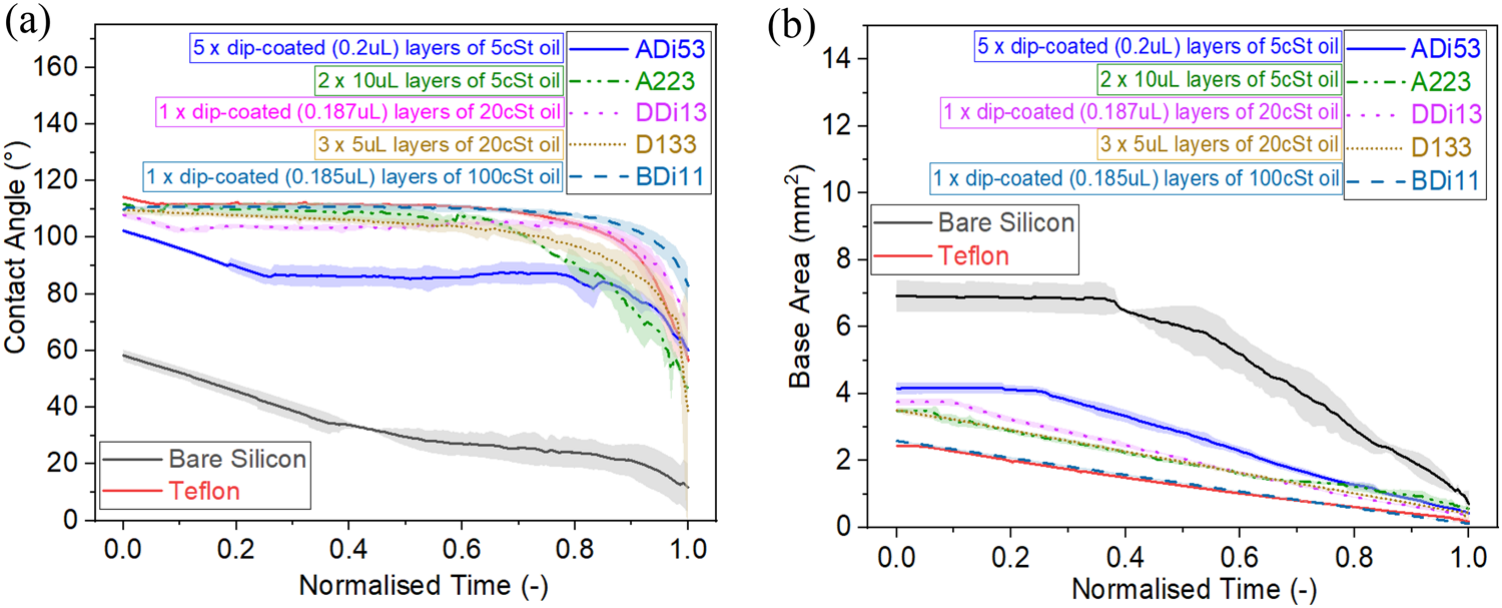
**(a)** Contact angle and (°) and **(b)** base area (mm^2^) as function of normalised time (−) during 4 µL water droplet evaporation on the different substrates fabricated via the parameters reported in the legend. Solid, dashed or dotted lines represent the mean values of three experimental runs, while the shading area above and below the average lines indicates the standard deviation. The results of water droplet evaporation on bare silicon and Teflon are additionally included for comparison^20^.

Figure 8a and Fig. 8b represent the contact angle and base area respectively versus normalised time as a water droplet evaporates. From Fig. 8 it is clear that the evaporation behaviours on all grafted surfaces, independently of the oil deposition method applied, lie within the behaviours reported for silicon and Teflon, both in terms of contact angle and base area trends. Since the wettability of our oil grafted surfaces is hydrophobic, all the initial contact angles reported are greater than 90° and the pinning time or CCR mode duration is greatly reduced when compared to the intrinsically hydrophilic bare silicon surface with an evaporation behaviour closer to the one reported on Teflon.

When looking in detail into Fig. 8, samples grafted with 100 cSt high viscosity oil (BDi11) droplets show lowest/negligible initial CCR mode mainly evaporating in the CCA mode. This behaviour is very much similar to that on Teflon coated samples, which will be quantified in terms of evaporation modes durations within the next subsection. In addition, from Fig. 8 it is evident that 20 cSt medium viscosity oil grafted sample prepared by multiple number of layers via the pipette method such as D133 shows similar behaviour to that observed for one layer 20 cSt medium and 100 cSt high viscosity grafted oils via the dip coating method of one single layer such as DDi13 and BDi11. Moreover, it is relevant to also highlight that as the viscosity of the oil and the volume/number of layers of grafted oil decreases such as for ADi53 dip-coated sample, then longer initial pinning/CCR mode ensues and the evaporation behaviour resembles that on bare silicon albeit with higher contact angles as can be clearly seen in Fig. 8a, i.e., ADi53 vs. silicon.

**Figure 9:**
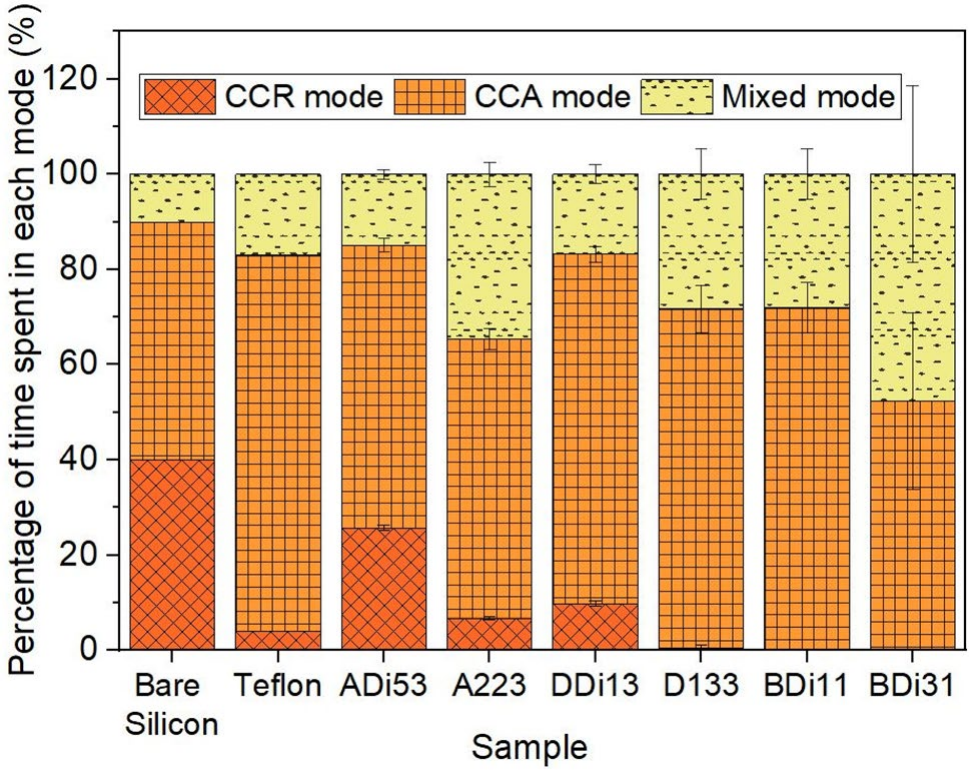
Percentage of normalised time (%) spent in the constant contact radius (CCR), constant contact angle (CCA) and mixed modes during droplet evaporation on bare silicon, Teflon, 5 layers of 5 cSt oil via 0.2 µL dip-coating deposition ADi53, 2 layers of 5 cSt oil via 10 µL pipette deposition A223, 1 layer of 20 cSt oil via 0.187 µL dip-coating deposition DDi13, 3 layers of 20 cSt oil via 5 µL pipette deposition D113, 1 layer of 100 cSt oil via 0.185 µL dip-coating deposition BDi11 and 3 layers of 100 cSt oil via 0.185 μL dip-coating deposition BDi31.

Next, Fig. 9 compares the normalised time spent in each evaporation mode for six representative grafted samples as well as for bare silicon and Teflon, which have been extracted from Fig. 8. On one hand, when looking at the two extremes, on uncoated bare silicon, up to 40% of the evaporation initially ensues in the CCR mode, followed by around 50% of evaporation in the CCA mode and the remaining of the evaporation ~ 10% ensuing in the mixed mode, which is consistent with earlier works^20^. While on smooth hydrophobic Teflon, only a short duration of the CCR mode of approximately 4% ensues while most of the droplet evaporates following the CCA mode for approximately 79% of the droplet lifetime, with the mixed mode ensuing at the end of the evaporation for about 17%^20^. On the other hand, silicone oil grafted samples offer lower initial pinning/CCR mode duration when compared to bare hydrophilic silicon, which can be tuned between < 1% to 25%. The CCR mode is then followed by the CCA mode ensuing thereafter for 55% to 75% of the droplet lifetime, and evaporation concludes in the mixed mode, which can also be varied between 15% to 45% of the droplet evaporation lifetime. While on one layer dip-coated 100 cSt BDi11 substrate there is almost complete suppression of the initial pinning/CCR stage where the CCR mode ensues for < 1% of total evaporation time. Additionally, the percentage of the time spent in the CCA mode was greatly increased with some substrates exhibiting > 70% of total droplet lifetime in this evaporation mode when compared to 50% or less in the case of bare silicon. When comparing our dip-coated one layer 100 cSt oil grafted BDi11 sample with the benchmarked Teflon, we are able to decrease the initial pinning time from 4% down to < 1% while maintaining the CCA mode in the same range as ~ 72%. Moreover, when comparing dip-coated five layers 5cSt oil sample ADi53 to bare silicon, a decrease in the initial pinning/CCR mode from 40% to < 30% and a slight increase in the subsequent CCA mode from 50% to 55–60% and in the mixed mode from 10 to 15% is further achieved and reported.

Besides comparing the different evaporation regimes and durations amongst our own fabricated samples reported in Figs. 3, 4, 5, and 8, additional comparison with the state-of-the-art pinning-free SOCAL surfaces reported in the work of Armstrong et al.^21^ is also carried out. In their work, water droplets on the same SOCAL surface at 40 ± 10% relative humidity initially evaporate in the mixed mode where both contact angle and contact area decrease for the first 10% of the evaporation time following a relaxation time, as opposed to initial CCR evaporation mode reported here. Then the classic CCA mode of evaporation ensues for the majority of the evaporation time (68 ± 1%) at a constant contact angle of 104.5 ± 0.4°. A second mixed mode is then reported for the final 21 ± 1% of the evaporation time where the contact area continues to decrease and the contact angle decreases swiftly until the droplet completely vanishes. Evaporation behaviour on SOCAL surfaces is then comparable to the dip-coated one layer 100 cSt oil sample BDi11, which exhibits droplet evaporation in the CCA mode for 72 ± 5% of the total evaporation time with a rather negligible initial pinning/CCR and/or mixed mode taking place for less than 1% at the beginning of the evaporation. Though to note is the easier and scalable fabrication procedure of BDi11 when compared to SOCAL surfaces.

Apart from these SOCAL surfaces, SLIPSs have also been investigated thoroughly in the literature aiming to minimise droplet contact line pinning^25^. In the work of Guan et al., the constant contact angle CCA mode ensues during most of the droplet lifetime as per the linearity of the contact area function of time, although the results reported do not allow to infer whether the first stage and the latest stage of the droplet evaporation occurs in the mixed mode as in SOCAL surfaces^25^. Recently Üçüncüoğlu and Erbil have investigated the droplet evaporation behaviour on SLIPSs with three different types of porous patterns, created via deep reactive ion etching, further impregnated with 20 cSt and 350 cSt silicone oils^27^. In addition to the different viscosity of the lubricant impregnated or infused, other varying parameters addressed were the lubricant thickness and the ridge height. For comparison, we have selected four samples from their work (two impregnated with 20 cSt oil and two with 350 cSt oil) for which droplets showed the CCA mode of evaporation for more than 40% of their evaporation lifetime. After analysing the images using imageJ^66^ and normalizing the time, the highest CCA mode duration occurs for the sample impregnated with 350 cSt oil with circular patterns geometry (40 µm size and 40 µm spacing, sample S3). This sample showed approximately no initial CCR mode and spent ~ 71 ± 1% time in CCA mode followed by ~ 28 ± 1% mixed mode of evaporation. This evaporation behaviour is very much in line with our dip-coated one layer 100 cSt oil sample BDi11, which completely supresses the initial CCR mode and spending ~ 72 ± 5% of time in CCA mode of evaporation (Fig. 5). Looking further into our 100 cSt grafted samples, it is worth noting that despite the similar trends observed where the initial CCR mode is supressed, the time spent in the CCA mode decreases with increasing the number of layers from 75% for BDi11 to 55% for BDi31, which is 20% less time in the CCA mode when increasing the number of layers from 1 to 3. The decrease in the CCA mode duration occurs in favour of a longer mixed mode at the end of the evaporation caused by the consequent grafting of the silicone oil onto the already grafted first layer, which slightly increases roughness and/or heterogeneities on the coating. This lower duration of the CCA mode is comparable to the 20 cSt oil with circular patterns geometry (40 µm size and 40 µm spacing, sample S3), which spends 60 ± 1% of the droplet lifetime in the CCA mode. These results are comparable in terms of evaporation modes and durations; however, SLIPSs require several manufacturing steps including micro-/nano-structure fabrication, deposition of a conformal hydrophobic coating and the further impregnation of the oil; whereas a single fabrication step is required in the case of the dip-coated one layer 100 cSt oil sample BDi11. In addition to the more complex fabrication procedure, SLIPS are often infused with excess of lubricant and suffer from oil depletion, whereas our fabrication method does not require any further impregnation step. The potential for grafted surfaces to empower similar evaporation behaviours to those reported on SLIPSs is then highlighted.

An important detailed observation on our silicone oil coated surfaces during the evaporation in what appears to resemble the CCA mode of evaporation is the occurrence of a distinctive stick–slip behaviour taking place when compared to the benchmarked smooth Teflon surface. As earlier reported in Fig. 2b, droplets on Teflon evaporate following the smooth receding of the contact line or base area in the absence of stick–slip with a rather constant contact angle CCA during most of the droplet lifetime. Meanwhile small discrete pinning and de-pinning events of the contact line and changes in contact angle in a stick–slip fashion occur on silicone oil grafted samples prepared with small volumes of low viscosity oils and low number of layers and/or high volumes of medium/high viscosity oils and high number of layers.

**Figure 10:**
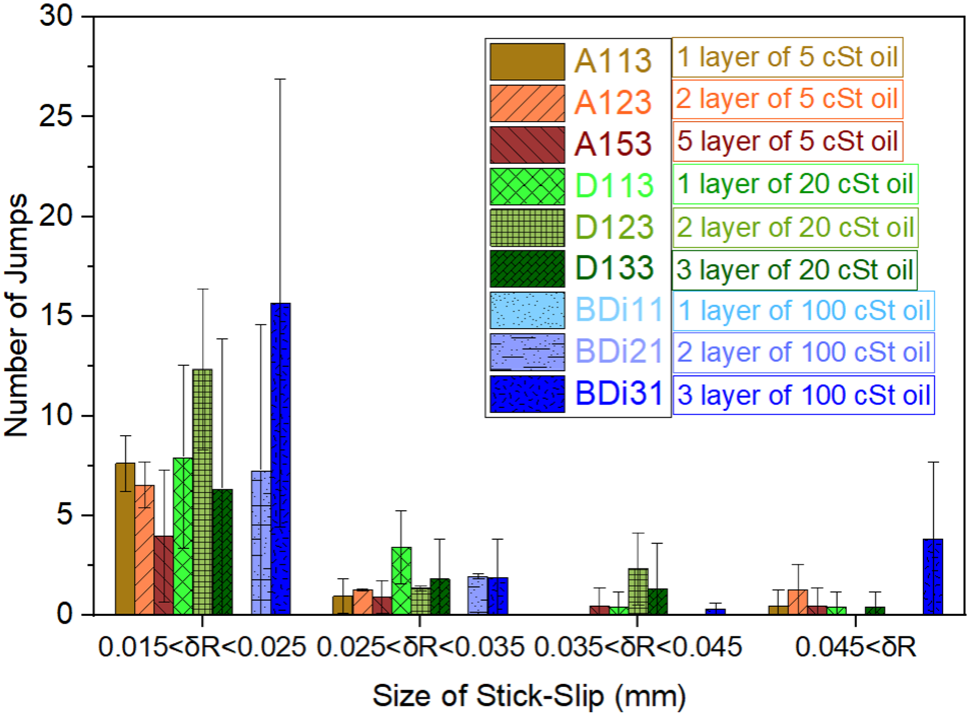
Stick–slip number of jumps during droplet evaporation on silicione oil grafted samples as a function of the magnitude of the jumps grouped in intervals of 0.01 mm between 0.015 mm and 0.025 mm, 0.025 mm and 0.035 mm, 0.035 mm and 0.045 mm, and greater than 0.045 mm. A total of 9 representative samples are presented, 3 from each viscosity oil used (5 cSt, 20 cSt and 100 cSt). 5 cSt and 20 cSt oil grafted samples are prepared via the pipette method while100 cSt oil grafted samples are prepared via dip-coating. Error bars represent the standard deviation of the number of jumps from three independent experiments.

Next, we further look into the different finite stick–slip jumps of the contact line during droplet evaporation. Figure 10 then provides the relevant quantification on the number of jumps as a function of the magnitude/size of the stick–slip jump, *x* (mm), by making use of 9 representative samples, 3 from each viscosity oil used (5 cSt and 20 cSt oil grafted samples prepared the via pipette method and 100 cSt oil grafted samples prepared via dip-coating). For simplicity, we group these stick–slip events in intervals of 0.01 mm between 0.015 mm and 0.025 mm, 0.025 mm and 0.035 mm, 0.035 mm and 0.045 mm and greater than 0.045 mm.

From Fig. 10, it can be seen that, for low viscosity 5 cSt oil grafted samples, the number of jumps decreases for the first interval (0.015 < *x* < 0.025) as the number of layers increases from 1 to 5. This proves that as the number of layers increases the full coating coverage and hence less likelihood for contact line pinning is guaranteed. Whereas for larger intervals, when comparing the different number of layers, the number of jumps is comparable and within the standard deviation bounds. Similar behaviour is observed on 20 cSt oil grafted samples in terms of the magnitude of the jumps. However, the number of jumps is somewhat higher than for 5 cSt grafted oil when comparing the same number of grafted layers, which may be due to surface heterogeneities arising from the subsequent grafting of medium/high viscosity oil layers containing larger molecules, which also increased the initial pinning/CCR mode. When looking at 100 cSt oil grafted samples, it is evident that dip-coated one layer 100 cSt oil sample BDi11 is able to suppress any stick–slip behaviour within the observable range, which resembles the evaporation behaviour on pinning-free SOCAL surfaces and benchmarked Teflon earlier reported in the literature. Meanwhile, as the number of layers increases for 100 cSt oil grafted samples, both the number as well as the length of the jumps also increase. As discussed in an earlier subsection (effect of grafted oil viscosity) and above, one layer of high viscosity grafted oil deposited via dip-coating results in a smooth surface providing full coverage and almost no pinning sites. However, when a second and/or a third layer are grafted on top of the first layer, the surface heterogeneities increase, which impose discrete droplet pinning and/or stick–slip behaviour during evaporation giving rise to higher number and greater magnitude of the stick–slip jumps. Overall, from Fig. 10, it can be observed that the number of stick–slip events for all lengths/intervals decreases as the number of layers of low viscosity oil grafted (5 cSt) increases. Whereas an opposite behaviour is evident in the case of 100 cSt high viscosity oil grafted samples where a decrease in the number of stick-slips events ensues as the number of layers decreases.

When comparing the stick–slip results reported in this work in Fig. 10 with those available in the literature, the magnitude of the stick–slip jumps are in agreement with those observed during the evaporation of low concentration 0.001% and 0.01% TiO_2_-water nano-fluids where changes in the base diameter ranged between 0.01 and 0.05 mm^20,67^. Similar values on the magnitudes of the stick–slip events on micro-structured SLIPSs have also been found in the work of Üçüncüoğlu and Erbil where localised pinning and de-pinning for evaporating water droplets on squared micro-structures' tops was noticed when the lubricant level is less than the micro-structures height.^27^. Moreover, Smith et al. also reported the occurrence of droplet pinning at the microstructures’ tops for lubricant levels lower than the height of the micro-structures^23^. However, they were able to minimise pinning at the microstructures’ tops by adding nanoscale roughness.

Since the pinning and de-pinning of the contact line during stick–slip occurrence is related to the excess of free energy gained by a droplet during evaporation in the pinning regime δ*G*; next we make use of the mathematical model for spherical cap droplets proposed by Shanahan in 1995 so to quantify the excess free energy during the different stick–slip events^68^. In the present case, the high mobility of the contact line coupled with the small changes in both contact angle and contact radius support the spherical cap assumption conveyed during the Methods section as well as the use of the mathematical model proposed by Shanahan. The droplet excess of surface free energy δ*G* for the different coated samples during the different stick–slip events can be either quantified by making use of Eqs. (3) and/or (4)^20,68,69^:

3 $$\updelta G= \frac{\gamma {sin}^{2}{\theta }_{ss} (2+{\text{cos}}{\theta }_{ss})(\updelta {R)}^{2}}{2R}$$

4 $$\updelta G= \frac{\gamma R{(\delta \theta )}^{2}}{2(2+{\text{cos}}{\theta }_{ss})}$$

where δ*R* is the magnitude of the stick–slip jumps reported in Fig. 10, while δ*θ* represents the change in contact angle, *R* is the droplet radius, and *θ*_ss_ is to the contact angle at which the stick–slip behaviour evolves, which can be extracted from the different droplet evaporation profiles.

**Figure 11:**
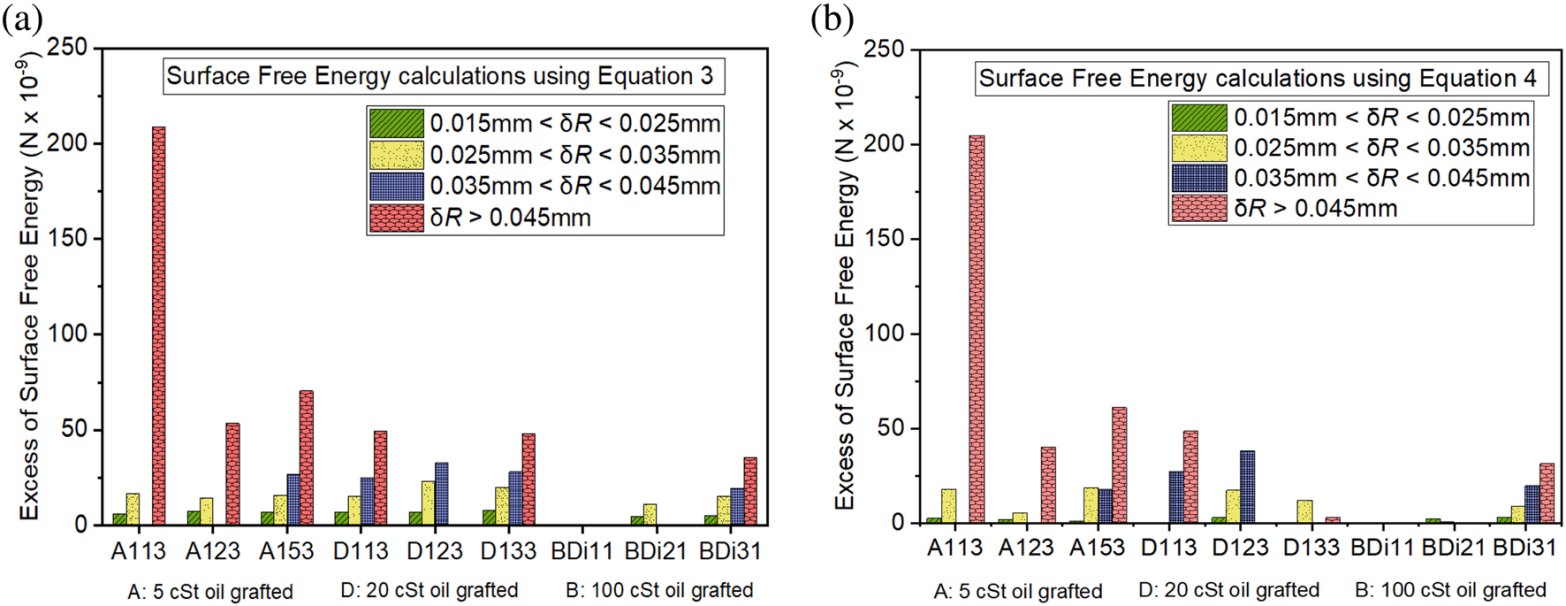
Excess of surface free energy, δ*G* (N), gained by a droplet during a stick–slip event represented within the four set stick–slip intervals for 9 representative samples: 3 from each viscosity oil used (5 cSt, 20 cSt and 100 cSt), using **(a)** Eq. (3) and **(b)** Eq. (4). 5 cSt and 20 cSt oil grafted samples are prepared via the pipette method while 100 cSt oil grafted samples are prepared via the dip-coating method. Samples represented here are the same as the ones reported in Fig. 10.

On one hand, Eq. (3) estimates the droplet excess of free energy by looking at the changes in contact radius after a depinning event while Eq. (4) makes use of the changes in contact angle during a pinning event. The surface free energy δ*G* by making use of either Eqs. (3) or Eq. (4) are represented in Figs. 11a and 11b respectively: When looking at the results presented in Fig. 11, since the mathematical equations developed by Shanahan, i.e., Eq. (3) and Eq. (4)*,* are equivalent^20,68^, a good quantitative agreement is found independently of the equation used with some minor exceptions^69^. In addition, since these equations are a function of the magnitude of the change in contact radius or contact angle, evidently greater free surface energies are estimated as the magnitude of the jumps increase. If we now focus on the different samples, the excess of surface free energy values are in the same order of magnitude ~ 10^–8^ except for 5 cSt oil grafted A113 sample, which reaches one order of magnitude higher values in the order of 10^–7^ N. As described in the previous section, this is due to the presence of pinning sites following an incomplete coating coverage during the oil grafting procedure. Quantitative values on the excess of surface energy calculated here are in agreement with those reported in Orejon et al. during the evaporation of low concentrations TiO_2_-water nano-fluids smaller than 0.05%^20^. On other hand, one layer 100 cSt oil grafted samples BDi11 shows no stick–slip behaviour during droplet evaporation with the consequent similar evaporation behaviour to that reported on the benchmarked smooth Teflon surface. Subsequently, after grafting a second and a third layer, some jumps are observed within the first two/shorter intervals and for all four intervals respectively, which is due to the small changes in roughness after higher viscosity oil evaporation during subsequent oil grafting steps as earlier reported in Abbas et al.^46^. Overall, the excess of surface free energy required for the contact line to de-pin during the stick–slip evaporation mode is in agreement with earlier works and obeys the following conditions; greater surface free energy is required for small volumes and small number of layers of low viscosity oils grafted; while lower excess of surface free energy is needed for smaller volumes and low number of layers for medium and high viscosity oils grafted; with 1 layer 100 cSt grafted sample BDi11 providing the lowest of the excess of surface energies reported.

## Conclusions

A simple one-step coating procedure by grafting silicone oil is able to hydrophobically modify the otherwise intrinsic hydrophilicity of the silicon surface. In this work, the evaporation dynamics and mechanisms of pure water droplets on silicone oil grafted surfaces adopting different fabrication parameters have been then investigated. The results show that all of the coatings empower the hydrophobic nature to the surfaces as well as the decrease in the contact angle hysteresis when compared to bare silicon. In addition, depending on the coating procedure adopted, the duration of the various evaporation modes such as the CCR mode where the contact line is pinned, CCA mode where the contact line recedes with a constant contact angle, and mixed mode where both contact angle and contact radius decrease, can be tailored to our advantage. More specifically, it is found that small volumes and low number of layers of high viscosity oil grafted increase the contact angle but more importantly decrease the contact angle hysteresis greatly, which allows pure water droplets to evaporate in the CCA mode for longer periods of time and with a rather similar behaviour to that on benchmarked Teflon. Similar observations are also reported when the volume or the number of layers of low viscosity oil are increased and these results are further supported by AFM analysis on these surfaces. Additionally, a discrete stick–slip behaviour of the contact line as droplets evaporate has been also observed, whose magnitude and number of occurrences depend on the fabrication procedure such as oil viscosity, number of layers, volume, and method of oil deposition prior to grafting, etc. Hence, simple and fast procedure of grafting silicone oil is effective in producing hydrophobic coatings on which the pinning time of evaporating droplets is greatly reduced and the contact line is able to recede smoothly for the majority of the droplets lifetime, as well as the tuning between the different evaporation regimes duration. These coatings have great potential for use in industrial applications such as self-cleaning surfaces and uniform particle deposition formation.

## Data Availability

The datasets used and/or analysed during the current study available from the corresponding author on reasonable request.

## References

[1] Uhlig BA, Wissemeier AH (2000). Reduction of non-ionic surfactant phytotoxicity by divalent cations. Crop Prot..

[2] Gibbons MJ, Di Marco P, Robinson AJ (2018). Local heat transfer to an evaporating superhydrophobic droplet. Int. J. Heat Mass Transf..

[3] Kita Y, Nakamatsu M, Hidaka S, Kohno M, Takata Y (2022). Quenching mechanism of spray cooling and the effect of system pressure. Int. J. Heat Mass Transf..

[4] Picknett RG, Bexon R (1977). The evaporation of sessile or pendant drops in still air. J. Colloid Interface Sci..

[5] Chen X, Ma R, Li J, Hao C, Guo W, Luk BL, Li SC, Yao S, Wang Z (2012). Evaporation of droplets on superhydrophobic surfaces: surface roughness and small droplet size effects. Phys. Rev. Lett..

[6] He M, Liao D, Qiu H (2017). Multicomponent droplet evaporation on chemical micro-patterned surfaces. Sci. Rep..

[7] Wang Z, Orejon D, Takata Y, Sefiane K (2022). Wetting and evaporation of multicomponent droplets. Phys. Rep..

[8] Fukatani Y, Orejon D, Kita Y, Takata Y, Kim J, Sefiane K (2016). Effect of ambient temperature and relative humidity on interfacial temperature during early stages of drop evaporation. Phys. Rev. E.

[9] Deegan RD, Bakajin O, Dupont TF, Huber G, Nagel SR, Witten TA (1997). Capillary flow as the cause of ring stains from dried liquid drops. Nature.

[10] Young III T (1997). An essay on the cohesion of fluids. Philos. Trans. R. Soc. Lond..

[11] Gresham IJ, Neto C (2023). Advances and challenges in slippery covalently-attached liquid surfaces. Adv. Colloid Interface Sci..

[12] McHale G, Gao N, Wells GG, Barrio-Zhang H, Ledesma-Aguilar R (2022). Friction coefficients for droplets on solids: The liquid–solid Amontons’ laws. Langmuir.

[13] de Gennes PG (1985). Wetting: Statics and dynamics. Rev. Mod. Phys..

[14] Hedestrand G (1924). On the influence of thin surface films on the evaporation of water. J. Phys. Chem..

[15] Rowan SM, Newton MI, McHale G (1995). Evaporation of microdroplets and the wetting of solid surfaces. J. Phys. Chem..

[16] Kordás K, Mustonen T, Tóth G, Jantunen H, Lajunen M, Soldano C, Talapatra S, Kar S, Vajtai R, Ajayan PM (2006). Inkjet printing of electrically conductive patterns of carbon nanotubes. Small.

[17] Köroğlu B, Lee KS, Park C (2013). Nano/micro-scale surface modifications using copper oxidation for enhancement of surface wetting and falling-film heat transfer. Int. J. Heat Mass Transf..

[18] Mampallil D, Eral HB (2018). A review on suppression and utilization of the coffee-ring effect. Adv. Colloid Interface Sci..

[19] Wang S, Wang Z, Li J, Li L, Hu W (2020). Surface-grafting polymers: From chemistry to organic electronics. Mater. Chem. Front..

[20] Orejon D, Sefiane K, Shanahan MER (2011). Stick-slip of evaporating droplets: Substrate hydrophobicity and nanoparticle concentration. Langmuir.

[21] Armstrong S, McHale G, Ledesma-Aguilar R, Wells GG (2019). Pinning-free evaporation of sessile droplets of water from solid surfaces. Langmuir.

[22] Lafuma A, Quéré D (2011). Slippery pre-suffused surfaces. Europhys. Lett..

[23] Smith JD, Dhiman R, Anand S, Reza-Garduno E, Cohen RE, McKinley GH, Varanasi KK (2013). Droplet mobility on lubricant-impregnated surfaces. Soft Matter.

[24] Wong T-S, Kang SH, Tang SKY, Smythe EJ, Hatton BD, Grinthal A, Aizenberg J (2011). Bioinspired self-repairing slippery surfaces with pressure-stable omniphobicity. Nature.

[25] Guan JH, Wells GG, Xu B, McHale G, Wood D, Martin J, Stuart-Cole S (2015). Evaporation of sessile droplets on slippery liquid-infused porous surfaces (SLIPS). Langmuir.

[26] Goodband SJ, Armstrong S, Kusumaatmaja H, Voïtchovsky, K. (2020). Effect of ageing on the structure and properties of model liquid-infused surfaces. Langmuir.

[27] Üçüncüoğlu R, Erbil HY (2023). Water drop evaporation on slippery liquid-infused porous surfaces (SLIPS): Effect of lubricant thickness, viscosity, ridge height, and pattern geometry. Langmuir.

[28] Wilson SK, D'Ambrosio H-M (2023). Evaporation of sessile droplets. Annu. Rev. Fluid Mech..

[29] Erbil HY (2012). Evaporation of pure liquid sessile and spherical suspended drops: A review. Adv. Colloid Interface Sci..

[30] Cazabat A-M, Guéna G (2010). Evaporation of macroscopic sessile droplets. Soft Matter.

[31] Larson RG (2014). Transport and deposition patterns in drying sessile droplets. AIChE J..

[32] Erbil HY, McHale G (2023). Droplet evaporation on superhydrophobic surfaces. Appl. Phys. Lett..

[33] Chen L, Huang S, Ras RHA, Tian X (2023). Omniphobic liquid-like surfaces. Nat. Rev. Chem..

[34] Eifert A, Paulssen D, Varanakkottu SN, Baier T, Hardt S (2014). Simple fabrication of robust water-repellent surfaces with low contact-angle hysteresis based on impregnation. Adv. Mater. Interfaces.

[35] Abdulkareem AA, Abusrafa E, Zavahir S, Habib S, Sobolčiak P, Lehocky M, Pištěková H, Humpolíček P, Popelka A (2022). Novel slippery liquid-infused porous surfaces (SLIPS) based on electrospun polydimethylsiloxane/polystyrene fibrous structures infused with natural blackseed oil. Int. J. Mol. Sci..

[36] Anuzyte E, Vaisis V (2018). Natural oil sorbents modification methods for hydrophobicity improvement. Energy Proc..

[37] Samyn P (2013). Wetting and hydrophobic modification of cellulose surfaces for paper applications. J. Mater. Sci..

[38] Shao F, Cao J, Ying Y, Liu Y, Wang D, Guo X, Wu Y, Wen Y, Yang H (2020). Preparation of hydrophobic film by electrospinning for rapid SERS detection of trace triazophos. Sensors.

[39] Dash S, Garimella SV (2013). Droplet evaporation dynamics on a superhydrophobic surface with negligible hysteresis. Langmuir.

[40] Wang L, McCarthy TJ (2016). Covalently attached liquids: Instant omniphobic surfaces with unprecedented repellency. Angew. Chem. Int. Ed..

[41] Jenkins A, Wells GG, Ledesma-Aguilar R, Orejon D, Armstrong S, McHale G (2023). Suppression of crystallization in saline drop evaporation on pinning-free surfaces. J. Chem. Phys..

[42] Bérard A, Patience GS, Chouinard G, Tavares JR (2016). Photo initiated chemical vapour deposition to increase polymer hydrophobicity. Sci. Rep..

[43] Cheng AKH, Soolaman DM, Yu H-Z (2006). Evaporation of microdroplets of ethanol−water mixtures on gold surfaces modified with self-assembled monolayers. J. Phys. Chem. B.

[44] Kobina Sam E, Kobina Sam D, Lv X, Liu B, Xiao X, Gong S, Yu W, Chen J, Liu J (2019). Recent development in the fabrication of self-healing superhydrophobic surfaces. Chem. Eng. J..

[45] Manoudis PN, Karapanagiotis I, Tsakalof A, Zuburtikudis I, Panayiotou C (2008). Superhydrophobic composite films produced on various substrates. Langmuir.

[46] Abbas A, Wells GG, McHale G, Sefiane K, Orejon D (2023). Silicone oil-grafted low-hysteresis water-repellent surfaces. ACS Appl. Mater. Interfaces.

[47] Hwang IG, Kim JY, Weon BM (2017). Droplet evaporation with complexity of evaporation modes. Appl. Phys. Lett..

[48] Al Balushi KM, Duursma G, Valluri P, Sefiane K, Orejon D (2023). Binary mixture droplet evaporation on microstructured decorated surfaces and the mixed stick-slip modes. Langmuir.

[49] Oh J, Orejon D, Park W, Cha H, Sett S, Yokoyama Y, Thoreton V, Takata Y, Miljkovic N (2022). The apparent surface free energy of rare earth oxides is governed by hydrocarbon adsorption. iScience.

[50] Tang X, Yan X (2017). Dip-coating for fibrous materials: Mechanism, methods and applications. J. Sol-Gel Sci. Technol..

[51] Launay, G. *PyDSA: Drop Shape Analysis in Python.* https://framagit.org/gabylaunay/pyDSA_gui (2018).

[52] Schofield FGH, Wilson SK, Pritchard D, Sefiane K (2018). The lifetimes of evaporating sessile droplets are significantly extended by strong thermal effects. J. Fluid Mech..

[53] Stauber JM, Wilson SK, Duffy BR, Sefiane K (2015). Evaporation of droplets on strongly hydrophobic substrates. Langmuir.

[54] Pan Z, Dash S, Weibel JA, Garimella SV (2013). Assessment of water droplet evaporation mechanisms on hydrophobic and superhydrophobic substrates. Langmuir.

[55] Darhuber AA, Troian SM, Davis JM, Miller SM, Wagner S (2000). Selective dip-coating of chemically micropatterned surfaces. J. Appl. Phys..

[56] Teisala H, Baumli P, Weber SAL, Vollmer D, Butt H-J (2020). Grafting silicone at room temperature—A transparent, scratch-resistant nonstick molecular coating. Langmuir.

[57] Chae SS, Jung JH, Choi WJ, Park JK, Baik HK, Jung J, Ko HW (2019). Multilayer fabrication of unobtrusive poly(dimethylsiloxane) nanobrush for tunable cell adhesion. Sci. Rep..

[58] Barca F, Caporossi T, Rizzo S (2014). Silicone oil: Different physical proprieties and clinical applications. BioMed. Res. Int..

[59] Steel DHW, Wong D, Sakamoto T (2021). Silicone oils compared and found wanting. Graefe's Arch. Clin. Exp. Ophthalmol..

[60] Chen Y, Askounis A, Koutsos V, Valluri P, Takata Y, Wilson SK, Sefiane K (2020). On the effect of substrate viscoelasticity on the evaporation kinetics and deposition patterns of nanosuspension drops. Langmuir.

[61] Ally J, Vittorias E, Amirfazli A, Kappl M, Bonaccurso E, McNamee CE, Butt H-J (2010). Interaction of a microsphere with a solid-supported liquid film. Langmuir.

[62] Dehnert M, Magerle R (2018). 3D depth profiling of the interaction between an AFM tip and fluid polymer solutions. Nanoscale.

[63] Mate CM, Lorenz MR, Novotny VJ (1989). Atomic force microscopy of polymeric liquid films. J. Chem. Phys..

[64] Gresham IJ, Lilley SG, Nelson ARJ, Koynov K, Neto C (2023). Nanostructure explains the behavior of slippery covalently attached liquid surfaces. Angew. Chem. Int. Ed..

[65] Krumpfer JW, McCarthy TJ (2011). Rediscovering silicones: “Unreactive” silicones react with inorganic surfaces. Langmuir.

[66] Rasband, W.S. *ImageJ*. https://imagej.nih.gov/ij/download.html. (National Institutes of Health, 2023).

[67] Orejon, D. *Study of Nanosuspension Droplets Free Evaporation and Electrowetting*. 175 (The University of Edinburgh, 2013).

[68] Shanahan MER (1995). Simple theory of "stick-slip" wetting hysteresis. Langmuir.

[69] Oksuz M, Erbil HY (2014). Comments on the energy barrier calculations during “stick–slip” behavior of evaporating droplets containing nanoparticles. J. Phys. Chem. C.

